# Evaluating urine volume and host depletion methods to enable genome-resolved metagenomics of the urobiome

**DOI:** 10.21203/rs.3.rs-4688526/v1

**Published:** 2024-08-05

**Authors:** Zachary J. Lewis, Angela Scott, Christopher Madden, Dean Vik, Ahmed A. Zayed, Garrett J. Smith, Sheryl S. Justice, Adam Rudinsky, Jessica Hokamp, Vanessa L. Hale

**Affiliations:** Department of Veterinary Preventive Medicine, The Ohio State University; Department of Veterinary Preventive Medicine, The Ohio State University; Department of Veterinary Preventive Medicine, The Ohio State University; Center of Microbiome Science, The Ohio State University; Department of Microbiology, The Ohio State University; Center of Microbiome Science, The Ohio State University; College of Nursing, The Ohio State University; Department of Veterinary Clinical Sciences, The Ohio State University; Department of Veterinary Biosciences, The Ohio State University; Department of Veterinary Preventive Medicine, The Ohio State University

**Keywords:** Urobiome, low biomass, genome-resolved metagenomics, host depletion, DNA extraction, Microbiome, Urine, canine

## Abstract

**Background:**

The gut microbiome has emerged as a clear player in health and disease, in part by mediating host response to environment and lifestyle. The urobiome (microbiota of the urinary tract) likely functions similarly. However, efforts to characterize the urobiome and assess its functional potential have been limited due to technical challenges including low microbial biomass and high host cell shedding in urine. Here, to begin addressing these challenges, we evaluate urine sample volume (100 ml – 5 mL), and host DNA depletion methods and their effects on urobiome profiles in healthy dogs, which are a robust large animal model for the human urobiome. We collected urine from seven dogs and fractionated samples into aliquots. One set of samples was spiked with host (canine) cells to model a biologically relevant host cell burden in urine. Samples then underwent DNA extraction followed by 16S rRNA gene and shotgun metagenomic sequencing. We then assembled metagenome assembled genomes (MAGs) and compared microbial composition and diversity across groups. We tested six methods of DNA extraction: QIAamp BiOstic Bacteremia (no host depletion), QIAamp DNA Microbiome, Molzym MolYsis, NEBNext Microbiome DNA Enrichment, Zymo HostZERO, and Propidium Monoazide.

**Results:**

In relation to urine sample volume, ^3^ 3.0 mL resulted in the most consistent urobiome profiling. In relation to host depletion, individual (dog) but not extraction method drove overall differences in microbial composition. DNA Microbiome yielded the greatest microbial diversity in 16S rRNA sequencing data and shotgun metagenomic sequencing data, and maximized MAG recovery while effectively depleting host DNA in host-spiked urine samples. As proof-of-principle, we then mined MAGs for core metabolic functions and environmental chemical metabolism. We identified long chain alkane utilization in two of the urine MAGs. Long chain alkanes are common pollutants that result from industrial combustion processes and end up in urine.

**Conclusions:**

This is the first study, to our knowledge, to demonstrate environmental chemical degradation potential in urine microbes through genome-resolved metagenomics. These findings provide guidelines for studying the urobiome in relation to sample volume and host depletion, and lay the foundation for future evaluation of urobiome function in relation to health and disease.

## Introduction

Alterations in the urobiome (microbiota of the urinary tract) have been associated with bladder cancer [1, 2], incontinence [3], urinary tract infection [4, 5], and urolithiasis [6, 7], but study of the urobiome remains challenging. Urine culturing is commonly employed to identify microbes and microbial functions (e.g., antimicrobial resistance) present in urine. However, standard urine culture captures very few members of the urobiota]. More recently, expanded quantitative urine culturing methods (EQUC) have improved culture resolution, but many urobiota remain uncultured, highlighting the need for effective culture-independent methods to profile the urobiota [8]. Critically, sequencing based studies of the urobiome are also fraught with technical challenges.

First, urine generally contains low microbial biomass [9], making urine samples vulnerable to contamination by microbes or microbial DNA introduced during extraction or sequencing [10, 11]. Additionally, there are no evidence-based guidelines on minimum urine volumes for microbiome research, and studies on the urobiome range from using 0.5 mL [12] to 50 mL of urine [13]. Importantly, there are conditions (e.g., urinary tract inflammation), populations (e.g., pediatric), and model species (e.g., dogs, rodents) for which collecting 10 mL of urine or more in a single void may be infeasible. Finally, urine can contain a high burden of host cells, especially in diseased states such as urinary tract infection or bladder cancer [14–16], which can complicate DNA extraction, introduce noise in 16S rRNA profiling [17] and overwhelm shotgun sequencing attempts with host reads rather than microbial reads. This then limits our ability to understand the functional potential of the urobiome and how these functions drive health and disease.

Commercial DNA extraction methods and published protocols that include host cell and DNA depletion are available, but these methods have not been comparatively evaluated in urine. In this study, we assess four commercially available DNA extraction kits that include host DNA depletion (MolYsis Complete5; NEBNext Microbiome DNA Enrichment Kit; QIAamp DNA Microbiome Kit; and Zymo HostZERO) as well as a protocol using light-activated propidium monoazide, and compare them to a method with no host depletion (BiOstic Bacteremia). Host depletion has been successful in other low-microbial-biomass, high-host-biomass substrates including breast milk, oral, respiratory tract, and tumor samples [18–22]. For example, in saliva samples, two host depletion methods reduced the host read proportion from 95% to < 30%, thereby improving the microbial resolution of shotgun metagenomics [19]. Host depletion methods offer promise for improving characterization of urobiome structure and function, but require evaluation for efficacy in urine samples.

The urobiome has been characterized via culture, whole genome sequencing of urine isolates, 16S rRNA gene sequencing, and shotgun metagenomic sequencing. However, few studies have reported metagenome-assembled microbial genomes (MAGs) and genome-resolved community analyses of the urobiota [23–26]. Bioinformatic construction of MAGs from urine would allow for more thorough functional reconstruction of the urobiome, including rare and unculturable taxa, revealing potentially important mechanistic links between the urobiome and disease in a genome-resolved fashion [27, 28].

In this study, we tested several approaches for studying the urobiome using urine from healthy dogs. Dogs are a robust translational model for the human urobiome[1, 29–31] and for urinary tract diseases, including bladder cancer [32] and urinary tract infection [29–31]. We specifically set out to **i**) assess the impact of urine sample volume on urine microbial community profiles (**Fig. S1**), **ii**) determine how DNA extraction methods that include host depletion affect urobiome profiles (16S rRNA and shotgun metagenomics) (**Fig. S2**), **iii**) determine if we could sufficiently reconstruct MAGs of urine microbes from shotgun metagenomic data to then mine them for relevant microbial functions, and **iv**) assess if and how urine microbes metabolize environmental chemicals linked with urinary tract diseases like bladder cancer.

## Methods

### Urine Volume Experiment

The goal of this first experiment was to determine if microbial community profiles and the presence/abundance of microbial contaminants (e.g. from reagents, kits, etc.) differed by urine sample volume (Experimental Design: **Fig. S1**).

### Subject Recruitment

Healthy dogs were recruited through the Ohio State University Veterinary Medical Center (IACUC: 2020A00000050). Each dog underwent a comprehensive physical exam, blood work (serum chemistry, complete blood count), urinalysis, and urine culture. All dogs were between one and ten years of age, weighed at least 20 lb with a body condition score of 4 or 5 (out of 9) and normal muscle condition. Dogs with a history, physical examination findings, clinical signs, or laboratory abnormalities consistent with urinary tract, liver, kidney, or gastrointestinal disease were excluded. Dogs with any history of antibiotic use, chemotherapy, or radiation in the past three months were also excluded (**Table S1**).

### Urine Sample Collection & Preparation

Midstream, free-catch urine was collected and stored from 5 healthy dogs as described previously [33]. Urine samples were fractionated into 0.1, 0.2, 0.5, 1.0, 3.0, and 5.0 mL aliquots prior to DNA extraction. Samples were centrifuged at 4°C and 20,000g for 30 minutes. Following centrifugation, supernatant was discarded, and the pellet was saved. The pellets were then used for DNA extractions.

### DNA Extraction & Quantification

DNA was extracted using the QIAamp BiOstic Bacteremia DNA Kit (Bacteremia; Qiagen, Hilden, Germany), as described previously [34]. This kit does not include host depletion steps. Briefly: pellets were resuspended in a lysis buffer and underwent two rounds of bead beating at 6m/s for 60s in an MP FastPrep-24 5G (MP Biomedicals, Solon, OH). Following bead beating, samples were cleaned using the kit’s inhibitor removal solution and processed according to manufacturer protocol. All centrifugation steps were conducted at 13,000 x g and, in the final step, samples were eluted twice through the silica membrane to maximize DNA yield. DNA concentrations were quantified using a Qubit Fluorometer (ThermoFisher Scientific, Waltham, MA).

### 16S rRNA Gene Library Preparation and Sequencing

DNA then underwent library preparation and sequencing at Argonne National Laboratory (Lemont, IL), as described previously [34]. Briefly: we used primers 515F and 806R to amplify the V4 region of the 16S rRNA gene, followed by paired-end amplicon sequencing via Illumina Miseq (2×250). Sequences are available at NCBI Bioproject PRJNA1109516.

### 16S rRNA Gene Sequence Processing and Statistical Analyses

Raw sequences were processed using QIIME2 v.2023–5. Reads were denoised and clustered into amplicon sequence variants (ASVs) using DADA2 [35]. with the following parameters: 5 base pairs (bp) were trimmed from the 5’ end of each read and forward reads were truncated at 225 bp while reverse reads were truncated at 220 bp. Putative contaminant reads were identified and removed using the R package *decontam* [36] with prevalence-based filtering (threshold = 0.5) (**Table S2**). Microbial contaminants are microbes or microbial sequences that get introduced during the extraction, library preparation, or sequencing process. These contaminants are putatively identified based on their tendency to be more prevalent or abundant in negative control samples (n = 7). Contaminant read counts were exported into a new table for analysis. Contaminant abundances were calculated by dividing each count by the total number of 16S rRNA reads in each sample, and contaminant abundances between groups were statistically compared using the Friedman test. Taxonomy was assigned using the Silva 138 99% OTU 515F/806R classifier. Unassigned sequences and sequences assigned to mitochondria or eukaryotes were removed. A total of 37 samples were sequenced, and sequencing depth (including negative controls) ranged from 1–30,408 reads. Samples with fewer than 4,125 reads were excluded from analyses, and remaining samples were rarefied to this depth. This excluded all negative controls and 6 true samples which were largely low volume samples (3 samples from dog ArB (0.1 ml, 0.5 ml, 3 ml), 1 sample from dog MS (0.1 ml), and two samples from dog FC (0.1 ml, 0.5 ml).

For all analyses, statistical significance was set at p < 0.05. Microbial diversity (Shannon Index, Observed Features, and Faith’s Phylogenetic Diversity) and distance metrics (Bray Curtis, Jaccard, and UniFrac) were calculated and tested using QIIME2 and the R packages *phyloseq*and *vegan*. Differences in bacterial diversity were assessed via t-test, Friedman test, or Kruskal-Wallis test depending on the normality and pairing of the data, and pairwise comparisons were conducted using the Benjamini, Krieger, and Yekutieli procedure for controlling the false discovery rate (FDR) at Q = 0.05 [37]. Differences in microbial composition were assessed via PERMANOVA, with Q = 0.05 for FDR adjustments in pairwise comparisons.

### Host Depletion – 16S rRNA Gene

The goal of this experiment was to evaluate how DNA extraction methods that include host depletion steps affected bacterial DNA recovery and microbial community profiles (Experimental Design: **Fig. S2**). Subject recruitment occurred as described above.

### Urine Sample Collection, Host Cell Spiking, & DNA Extraction

We collected mid-stream free catch urine from seven healthy dogs (**Table S1**). Urine was then aliquoted into two batches: one batch was spiked with canine cells (canine thyroid adenocarcinoma cells [38] – CTAC) to a concentration of 75,000 cells/mL, to model a biologically relevant host cell concentration in urine from healthy dogs [39]. The other batch remained unspiked. Urine samples were then pelleted as described above. All urine samples then underwent DNA extraction using six different extraction methods: QIAamp BiOstic Bacteremia DNA Kit (Bacteremia; Qiagen, Hilden, Germany); MolYsis Complete5 (Molzym, Bremen, Germany); NEBNext Microbiome DNA Enrichment Kit (New England Biolabs, Ipswich, MA); QIAamp DNA Microbiome Kit (Qiagen, Hilden, Germany); HostZERO Microbial DNA Kit (Zymo Research, Irvine, CA); and a protocol using light-activated propidium monoazide described in Marotz et al., 2018 [19] All of these methods except QIAamp Bacteremia included host depletion steps. In addition to urine samples, we also included a positive control sample (ZymoBIOMICS Gut Microbiome Standard, Zymo Research, Irvine, CA, **Table S3**) that we extracted with each method. The ZymoBIOMICS gut microbiome standard contains 21 microbial strains, including 18 bacteria, 2 microbial eukaryotes, and one archaeaon. Samples were extracted according to the respective manufacturers’ protocol, with modifications described below. Each extraction also included a negative control (blank) that underwent extraction and sequencing along with all the true samples (n = 6). All extracted DNA was stored at −80°C until library preparation and sequencing. Unspiked samples underwent 16S rRNA gene sequencing; spiked samples underwent shotgun metagenomic sequencing (described under Host Depletion – Shotgun Metagenomics).

### DNA Extraction Methods

#### QIAamp BiOstic Bacteremia (Qiagen)

No host depletion is included in this protocol. Protocol is detailed above under Urine Volume Experiment. For the ZymoBIOMICS Gut Microbiome Standard, prior to extraction, the standard was centrifuged at 20,000g for 10 minutes. The supernatant and pellet were then separated, and both were saved. Per recommendations from Zymo, the pellet was then processed through the Bacteremia kit following the manufacturer’s protocol. In the final step, the supernatant was added to the MB spin column (along with the pellet lysate) and centrifuged at 13,000g for 1 minute. This captured any additional DNA from the supernatant on the spin column

#### Molzym MolYsis Complete 5 (Molzym MoYsis)

This method uses a chaotropic buffer to selectively lyse host cells then removes host DNA using a DNAase prior to extracting microbial DNA. Samples were extracted following the manufacturer’s protocol.

#### NEBNext Microbiome DNA Enrichment Kit (NEBNext)

This method uses nonselective lysis followed by selective binding and depletion of CpG-methylated host DNA in order to enrich microbial DNA recovery. Samples were first extracted using the QIAamp BiOstic Bacteremia DNA Kit and frozen at −80°C. Samples were defrosted and further extraction was performed according to the NEBNext manufacturer’s protocol. For samples that did not have detectable DNA after the initial extraction, a threshold of 0.05 ng/μL was used to calculate the MBD2-Fc Protein to Protein A magnetic bead value. A solution of MBD2-FC Protein and Protein A magnetic beads was prepared and aliquoted into each sample accordingly. To avoid DNA loss, purification was not performed at the end of the protocol (neither option A nor B).

#### QIAamp DNA Microbiome Kit (DNA Microbiome)

This method uses selective osmotic lysis and Benzonase to degrade host cells and digest host DNA prior to extraction of microbial DNA. A Thermomixer at 600 rpm was used instead of end-over-end rotation. Prior to extraction, the ZymoBIOMICS Gut Microbiome Standard was centrifuged at 20,000g and the supernatant was saved. To maximize DNA recovery per recommendations from Zymo, the pellet was processed through the kit, and the supernatant was added to the MB spin column and centrifuged at 13,000g for 1 minute. The flow-through was discarded, and lysate from the pellet was added per the manufacturer’s protocol at step 12.

#### Zymo HostZERO Microbial DNA Kit (Zymo)

This method uses selective osmotic lysis followed by enzymatic degradation of DNA to degrade host cells and host DNA prior to extraction of microbial DNA. A FastPrep-24 5G bead beater was used for optimized lysis (Appendix D of manufacturer’s protocol). Extraction proceeded following the manufacturer’s protocol. Samples were eluted with 20–26 uL ZymoBIOMICs DNase RNase-Free Water.

#### Propidium Monoazide (PMA)

This method uses PMA to intercalate the DNA of membrane-disrupted host cells, and light activation triggers covalent bonding between dsDNA and PMA, fragmenting the DNA. Samples were pretreated with 10uM PMA as described in Marotz et al. (2018), beginning with resuspending urine pellets in 200uL sterile water. After PMA treatment, samples were stored at −20°C and then extracted using the Qiagen QIAamp BiOstic Bacteremia kit.

#### DNA Quanti**fi**cation and 16S rRNA Gene Sequencing

In both spiked and unspiked samples, we quantified total DNA via Qubit fluorometer and bacterial DNA via qPCR using universal 16S rRNA gene bacterial primers as described previously [34, 40]. Bacterial concentrations were compared between groups using either Friedman tests or Kruskal-Wallis tests. Finally, we analyzed microbial community profiles (16S rRNA gene sequencing) in each sample. Library preparation, sequencing, decontamination (**Table S4**), and analysis were conducted as described above in the Urine Volume Experiment with the following DADA2 parameters: 5 bp were trimmed from the 5’ end of each read and forward reads were truncated at 250 bp while reverse reads were truncated at 231 bp. One urine sample (Dog SJ, Extraction Method: Molzym Molysis) appeared to be cross-contaminated with DNA from the ZymoBIOMICS Gut Microbiome Standard and was excluded from analysis (**Fig S3**). Statistical analyses were performed as described above (see Urine Volume Experiment) to assess differences by extraction method.

#### 16S rRNA Gene Sequence Processing and Statistical Analyses

16S rRNA gene sequencing processing and statistical analyses were performed as described above (see Urine Volume Experiment) to assess differences in microbial community diversity and composition by extraction method.

#### Host Depletion – Shotgun Metagenomics

The goal of this experiment was to assess host depletion by extraction method and the viability of performing genome-resolved metagenomics on low biomass urine samples. To do this, we used the same urine samples and ZymoBIOMICs Gut Microbiome Standard positive control from the Host Depletion – 16S rRNA Gene experiment described above and spiked them with host (canine) cells (Experimental Design: **Fig. S2**). Spiking samples with an equal concentration of host cells allowed us to best assess the host DNA depletion efficacy of each method. DNA was extracted using the same 5 methods as described above under Host Depletion – 16S rRNA Gene (Bacteremia, DNA Microbiome, Molzym MolYsis, Propidium Monoazide, and Zymo HostZERO). NEBNext sequences did not pass quality control in 16S rRNA gene sequencing; we therefore excluded these samples from shotgun metagenomic sequencing.

#### Shotgun Metagenomic Library Preparation and Sequencing

Samples underwent shotgun metagenomic sequencing at the Ohio State University Infectious Diseases Institute – Genomics and Microbiology Solutions (IDI-GEMS) Laboratory. Metagenomic libraries were prepared following the Illumina (San Diego, CA) DNA Library Prep protocol with the following modifications: 1) Illumina’s (M) beads were substituted with (L) beads to obtain larger insert sizes, 2) 9 or 12 PCR amplification cycles were used based on sample DNA concentration (Qubit) (**Fig. S4**), and 3) library purification was performed using a 1:1 sample to bead ratio. Samples were barcoded using IDT for Illumina UD Indexes. Tagmentation-based library construction has been validated and adopted as a standard operating procedure within the IDI-GEMS Laboratory to characterize the presence of microbes in samples and was recently shown to be an effective repeatable method for microbiome analysis of the human gut [41]. Metagenomic libraries were sequenced targeting a minimum of 50 million 2×150 base pair paired-end reads using an Illumina NextSeq2000. Negative extraction (n = 5) and sequencing (n = 2) controls were sequenced along with samples. Sequences were processed using the Ohio Supercomputer [42]. Sequences are available at NCBI Bioproject PRJNA1123238.

#### Metagenomic Sequence Processing and Statistical Analyses

Raw reads from the Illumina sequencer were quality filtered and trimmed of adapters using Trimmomatic [43]. Host reads were quantified by mapping to a concatenated canine and feline genome with CoverM [44]. Reads not assigned host were assumed microbial. Read counts were compared across extraction methods using the Friedman test. Taxonomy and abundance tables for microbial community profiling of metagenomes were generated using MetaPhlAn4.0 [45] and SingleM [46] and SingleM condense. Metagenomes were *de novo* assembled into contigs using MEGAHIT [47] and quality assessed with QUAST [48]. Contigs were binned into MAGs using portions of the MetaWRAP [49] pipeline, which combines the binning methods MetaBat2 [50], MaxBin2 [51], and CONCOCT [52], and chooses the highest-quality representative of each bin from across these automated methods. dRep [53] was used to dereplicate MAGs at 99% average nucleotide identity, and CheckM [54] was used to evaluate MAGs for completeness and contamination. Only medium (> 70% completion and < 10% contamination) and high (> 95% completion and < 5% contamination) quality MAGs were retained for analysis. GTDB-Tk [55]. was used to assign taxonomy to MAGs according to the Genome Taxonomy Database. Abundance tables of MetaPhlAn, SingleM, and MAG profiles were processed using *decontam* to identify putative contaminants. Because MetaPhlAn generates species-level taxonomic assignments, genera were also manually filtered: taxa commonly identified as kit contaminant genera [56] present in at least one negative control sample were bioinformatically removed, even if they were not filtered by *decontam* (**Table S5**). Additionally, reads assigned to taxa from the Zymo Gut Microbiome Standard in urine samples profiled with MetaPhlAn or GTDB-Tk were considered putative cross-contaminants and were removed from those samples. Diversity and community composition metrics from metagenomic data as well as read-level statistics were analyzed using the R packages *phyloseq* [57], *vegan* [58], and *tidyverse* [59]. Alpha diversity was compared between kits using Friedman tests, and comparisons between dogs were performed using Kruskal-Wallis. Pairwise comparisons were conducted using the Benjamini, Krieger, and Yekutieli procedure for controlling the false discovery rate (FDR) at Q = 0.05. Differences in microbial composition were assessed via PERMANOVA, with Q = 0.05 for FDR adjustments in pairwise comparisons. Genes in MAGs were annotated using DRAM [27].

#### Hydrocarbon Degradation Pro**fi**ling

As a proof-of-principal test, we then mined the MAGs for microbial functions of interest including urea utilization and environmental chemical degradation. These functions are relevant as urine is a urea rich environment, and environmental chemicals, such as polycyclic aromatic hydrocarbons have been associated with urinary tract diseases like bladder cancer. Urea utilization was identified by searching within the DRAM output. To identify putative hydrocarbon degrading genes, we queried custom, curated, published Hidden Markov Model (HMM) profile databases: aerobic degradation of polycyclic aromatic hydrocarbon pathways (PAHp) [60], and markers for the activation of various hydrocarbons (CANT-HYD) [61]. Coding genes called by DRAM were queried against these databases using the hmmsearch function of HMMER (version 3.3) [62] and filtered to a maximum expect-value (e-value) of 1e-10. The full scores were compared to the score cutoffs specific to each gene in the database, i.e., gather cutoffs for PAHp and noise or trusted cutoffs implemented by CANT-HYD. Given the potential for high stringency in profiles generated largely from a few well-characterized model organisms, these cutoffs were relaxed to a minimum of 80% of the gather cutoff and 90% of the noise and trusted cutoffs for the respective databases.

## Results

### Urine Volume Experiment

**Figure 1:**
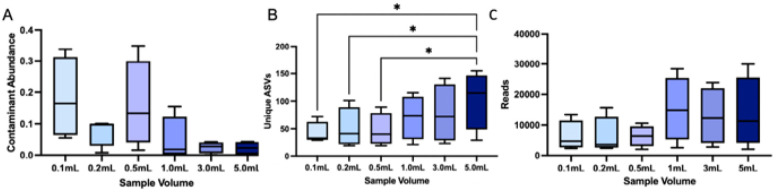
Urine sample volume influences contaminant abundance and microbial diversity (16S). A) The abundance of contaminants (contaminating microbial sequences) decreased significantly as sample volume increased (overall p=0.026, Friedman, no pairwise comparisons were significant, **Table S6**). B) Microbial richness, or the number of unique amplicon sequence variants (ASVs), increased significantly with increased sample volume (p=0.015, Friedman) and 5.0mL samples had significantly greater numbers of unique ASVs compared to 0.5mL (p=0.031), 0.2mL (p=0.031), and 0.1mL samples (p=0.048), (multiple comparisons were FDR-corrected at 0.05, **Table S7**). C) Sequencing depth (reads) was increased at greater urine sample volumes although this difference was not significant (p=0.075, Friedman). Box and whisker plots show the median, IQR, and min/max.

Current urobiome studies vary widely in the volume of urine used for profiling microbial communities. Moreover, low biomass samples, like urine, are highly susceptible to contamination by microbes or microbial DNA (hereafter referred to as “contaminants”) that can be introduced during the DNA extraction and sequencing process. As such, in this experiment, we first assessed the relationship between urine sample volume and microbial contaminant load. Contaminants, as identified by *decontam* (**Table S2**), were at significantly lower relative abundances in urine samples of greater volume (Fig. 1A, **Table S6**, *p* = 0.026, Friedman).

We then evaluated bacterial diversity and composition by urine sample volume. Microbial richness, or the total number of unique ASVs in each sample, increased significantly with sample volume (Fig. 1B, S5, **Table S7**, p = 0.015, Friedman). Sequencing reads also increased with urine sample volume; although, this difference was not significant (Fig. 1C, p = 0.075, Friedman).

**Figure 2:**
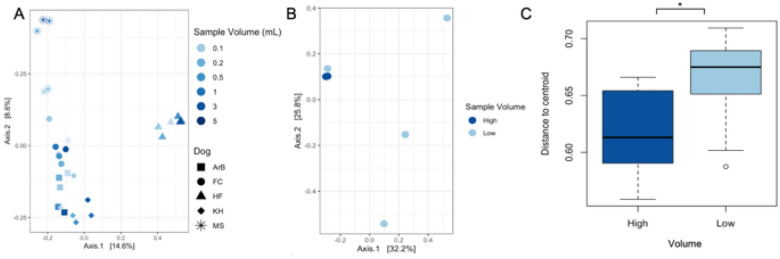
Urine sample volume and microbial composition (16S). A) Microbial composition (Bray-Curtis) of urine samples differed significantly by dog but not by sample volume (PERMANOVA: by dog p = 0.001; by sample volume p = 0.98). B) Representative Bray-Curtis plot of a single dog’s (Dog = MS) urine samples. C) High volume (≥ 3 mL) samples were significantly less variable (shorter distance to centroid) than low volume (≤ 1 mL) samples (Bray Curtis, p = 0.0017, PERMDISP).

Bacterial composition, however, did not differ significantly by urine sample volume but did differ significantly between dogs (Fig. 2A, S5, between dogs: *p* = 0.001, by urine sample volume: 0.98, Bray-Curtis, PERMANOVA), indicating that inter-dog differences overwhelmed differences based on sample volume. We next evaluated within-dog microbial composition by sample volume. Within each dog, the 3 mL and 5 mL samples were more consistent in microbial composition, while the 0.1, 0.2, 0.5 and 1 mL samples were more variable (Fig. 2B, S6). Based on this pattern, we grouped 3 mL and 5 mL urine samples into a “High” volume group, and the remaining urine volumes into a “Low” volume group. There was no significant difference in microbial composition between the High and Low groups (p = 0.6, PERMANOVA); however, High volume samples had significantly less variable microbial communities than Low volume samples, indicating that Low volume samples are more subject to stochasticity (Fig. 2C, S6; *p* = 0.0017, PERMDISP). Based on these results, we proceeded to use 3 mL urine samples for subsequent experiments.

### Host Depletion – 16S rRNA Gene

Healthy urine contains shed host epithelial cells at a relatively low abundance. However, in the presence of urinary tract disease (e.g., urinary tract infection, bladder cancer, bladder stones), host cell shedding can dramatically increase. There are multiple DNA extraction methods that incorporate host cell / host DNA depletion steps to facilitate microbial DNA recovery. In this experiment, we evaluated how six different extraction methods affected DNA concentrations and microbial community profiles. Extraction methods included: QIAamp BiOstic Bacteremia DNA Kit (Bacteremia); MolYsis Complete5 (Molzym); NEBNext Microbiome DNA Enrichment Kit; QIAamp DNA Microbiome Kit (DNA Microbiome) HostZERO Microbial DNA Kit (Zymo HostZERO); and a protocol using light-activated propidium monoazide described in Marotz et al., 2018 [19]. All methods except Bacteremia included host depletion steps. The Bacteremia extraction method was included for reference here because this method has already been validated as an optimal method for profiling canine urine microbial communitites [34], and it has been applied across multiple urobiome studies in humans and animals [1, 4]. However, it has not been tested against extraction methods that include host depletion steps, which we did here.

**Figure 3:**
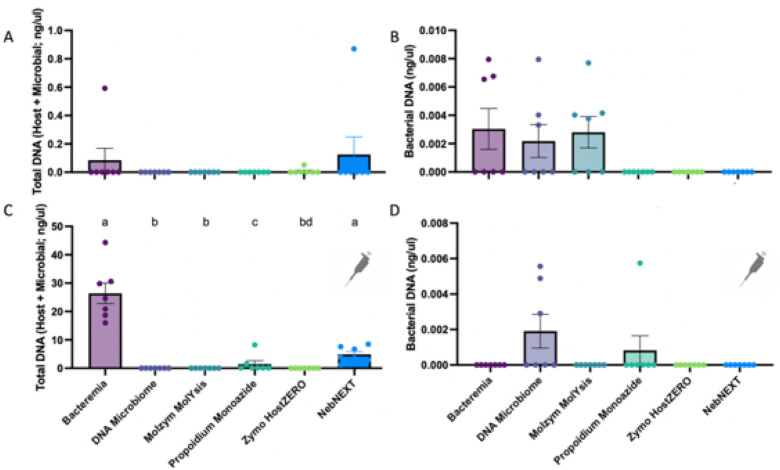
Total and Bacterial DNA recovery differed by extraction method. A) Total DNA concentrations (ng/ul, Qubit fluorometry) did not differ by extraction method (p=0.62, Friedman). B) Bacterial DNA concentrations (qPCR) differed significantly by extraction method (overall p=0.014, Friedman); although no pairwise comparisons were significant. C) Total DNA concentrations recovered from urine samples spiked with canine (CTAC) cells varied significantly by extraction method (p<0.0001, Friedman). Pairwise comparisons are indicated by letter above each bar. Bars with differing letters were significantly different (p<0.05, FDR 0.05). D) Bacterial DNA concentration from spiked urine samples marginally differed by extraction method (p=0.051, Friedman). Bars represent the mean with standard error. Pipettor icon in C) and D) indicates that all samples shown in these graphs were spiked with canine thyroid adenocarcinoma (CTAC) cells.

We first compared how each extraction method impacted total and bacterial DNA concentrations derived from urine samples. We also compared DNA concentrations in urine samples that were unspiked versus those spiked with host (canine) cells. While healthy mid-stream free-catch urine contains a low abundance of host cells, we opted to spike additional canine cells into urine at biologically relevant concentrations to best assess the host depletion capabilities of each extraction method. In unspiked samples, Bacteremia and NEBNext recovered the greatest total DNA concentrations (host + microbial); although, this result was not significant (p = 0.62, Friedman, Fig. 3A). Bacteremia, DNA Microbiome, and Molzym MolYsis demonstrated significantly greater *bacterial* DNA recovery than propidium monoazide, Zymo HostZERO, and NebNEXT; although no pairwise comparisons were significant (overall p = 0.014, Friedman, Fig. 3B). In spiked urine samples, Bacteremia and NebNEXT recovered significantly greater total DNA than all other extraction methods (Fig. 3C, overall p < 0.0001, Friedman, pairwise p between Bacteremia or NebNEXT and all other methods < 0.05), while DNA Microbiome recovered the most bacterial DNA; although, overall differences in bacterial DNA concentrations by extraction method were only marginally significant (Fig. 3D, overall p = 0.051, Friedman). There was no significant difference in total or bacterial DNA recovery by dog in unspiked or spiked samples (**Fig. S7**)

**Figure 4:**
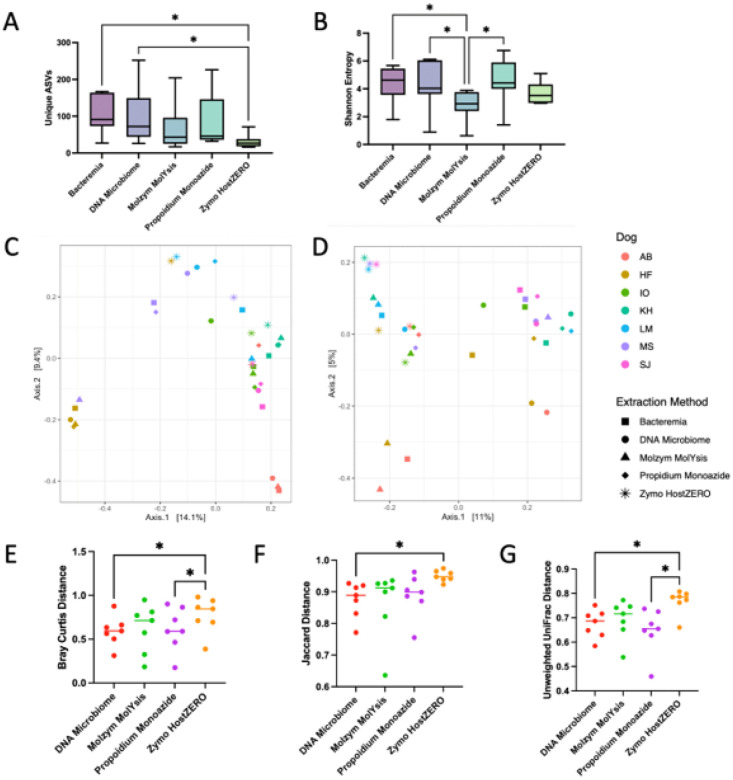
Microbial diversity and composition by extraction method (16S). A) Microbial richness, or number of unique ASVs, and B) Microbial diversity (Shannon entropy) differed significantly by extraction method (Richness p=0.0018, Shannon p=0.0091, Friedman, multiple comparisons with FDR at 0.05, **Table S8**). Whiskers represent minimum, maximum, and median. *p<0.05. C) Microbial composition (Bray-Curtis) differed significantly by dog (PERMANOVA p=0.001), but not extraction method (PERMANOVA p=0.92) D) When microbial composition was weighted by phylogeny (Unweighted UniFrac), composition differed significantly by both extraction method (PERMANOVA p=0.002), and by dog (PERMANOVA p=0.001). E) Bray-Curtis, F) Jaccard, and G) Unweighted UniFrac distances from Bacteremia-extracted samples to samples of the same dog extracted via other extraction methods. E) Bray-Curtis F) Jaccard, and G) Unweighted UniFrac distances differed significantly with DNA Microbiome samples being the most similar (shortest distance) to Bacteremia-extracted samples (Friedman, Bray-Curtis p=0.034; Jaccard p=0.0342; Unweighted UniFrac p=0.0071). Pairwise comparison p-values are outlined in **Table S9**. *p<0.05. Bar represents median.

We next assessed urine microbial diversity (16S rRNA) of unspiked urine samples by extraction method. Sequencing data from all samples extracted using NEBNext did not pass quality control steps [35] and, as such, were excluded from analysis. Urine microbial diversity varied significantly by extraction method (Fig. 4, S8, **Table S8**, Microbial richness p = 0.0018, Shannon Entropy p = 0.0091, Friedman). Specifically, urine samples extracted using Bacteremia and DNA Microbiome contained the greatest microbial richness (unique ASVs) and significantly greater microbial richness than samples extracted using Zymo HostZERO (Fig. 4A, **Table S8**, overall p = 0.0018, pairwise p = 0.0041, Friedman). Samples extracted via Bacteremia, DNA Microbiome, or propidium monoazide also exhibited the greatest microbial diversity (Shannon Entropy), all three showing significantly greater microbial diversity than samples extracted via Molzym MolYsis (Fig. 4B, **Table S8**, pairwise p = 0.025, 0.028, and 0.017, Friedman, respectively).

Finally, we assessed urine microbial composition (16S rRNA) of unspiked urine samples by extraction method. Microbial composition (Bray-Curtis) differed significantly by dog but not by extraction method (Fig. 4C, S8, Bray-Curtis, by dog p = 0.001, by kit = 0.92, PERMANOVA). When composition was weighted by phylogeny (relatedness of microbes between samples; Unweighted UniFrac), composition differed significantly by both extraction method and by dog (Fig. 4D, S8, Unweighted UniFrac, extraction method p = 0.002, dog p = 0.001, PERMANOVA). Urine samples extracted using Bacteremia, DNA Microbiome, and Molzym MolYsis exhibited more similar microbial composition as compared to samples extracted with propidium monoazide or Zymo HostZERO (Fig. 4E, F, G, **Table S9**, Bray-Curtis p = 0.037, Jaccard p = 0.034, Unweighted UniFrac p = 0.0071, Friedman).

### Host Depletion – Shotgun Metagenomics

**Figure 5:**
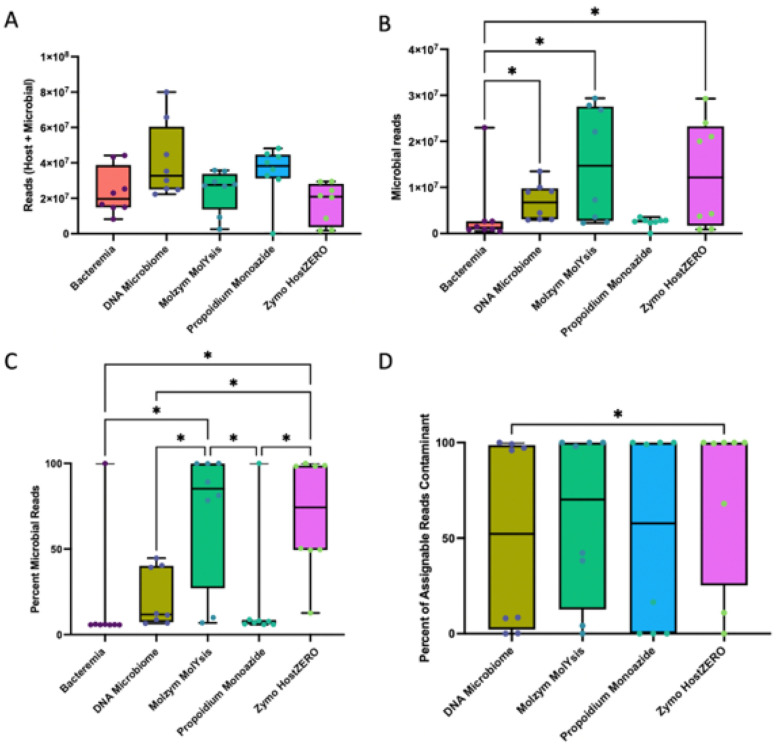
Extraction method impacted host and microbial read abundances (Shotgun metagenomics). A) Total sequencing reads did not vary by extraction method (p=0.12, Friedman). B) However, *microbial* reads did vary significantly by extraction method (p=0.0039, Friedman), with DNA Microbiome, Molzym MolYsis, and Zymo HostZERO exhibiting a greater number of microbial reads compared to Bacteremia (all pairwise p=0.01). C) The *proportion* of microbial reads also varied significantly by extraction method with Molzym MolYsis and Zymo HostZERO yielding the greatest proportion of microbial reads (overall p<0.0001, all pairwise p<0.02, Friedman). D) The abundance of contaminant reads also differed significantly by extraction method (overall Friedman p=0.014) and was lowest in DNA Microbiome (DNA microbiome vs. Zymo HostZERO pairwise p=0.01). (See **Table S5** for a list of contaminants).

We next assessed host depletion efficacy of each extraction method using shotgun metagenomic sequencing performed on urine samples spiked with host (canine) cells. Samples averaged 28.2 million paired-end reads per sample (range: 1399–80 million reads, SD: 16.7 million reads). There was no significant difference in the total number of reads obtained per sample by extraction method (Fig. 5A p = 0.12, Friedman). However, the total number of *microbial* reads did vary significantly by extraction method (Fig. 5B, p = 0.0039, Friedman), with DNA Microbiome, Molzym MolYsis, and Zymo HostZERO yielding a significantly greater number of microbial reads compared to Bacteremia, which includes no host depletion steps (all pairwise p = 0.01). The *proportion* of total microbial reads also varied significantly by extraction method with Molzym MolYsis and ZymoHostZERO yielding the greatest proportion of microbial reads (Fig. 5C, overall p < 0.0001, pairwise p < 0.02, Friedman). In terms of host reads, each method yielded the following (on average): Bacteremia, 82% host reads; DNA Microbiome, 78%; Molzym MolYsis, 29%; PMA, 81%; Zymo HostZERO, 30%. Finally, we quantified the abundance of contaminant reads by extraction method and found that DNA Microbiome samples contained the lowest abundance of contaminant reads (Fig. 5D, overall p = 0.014, Friedman), although contaminant read abundances varied widely between samples (0–100%).

**Figure 6:**
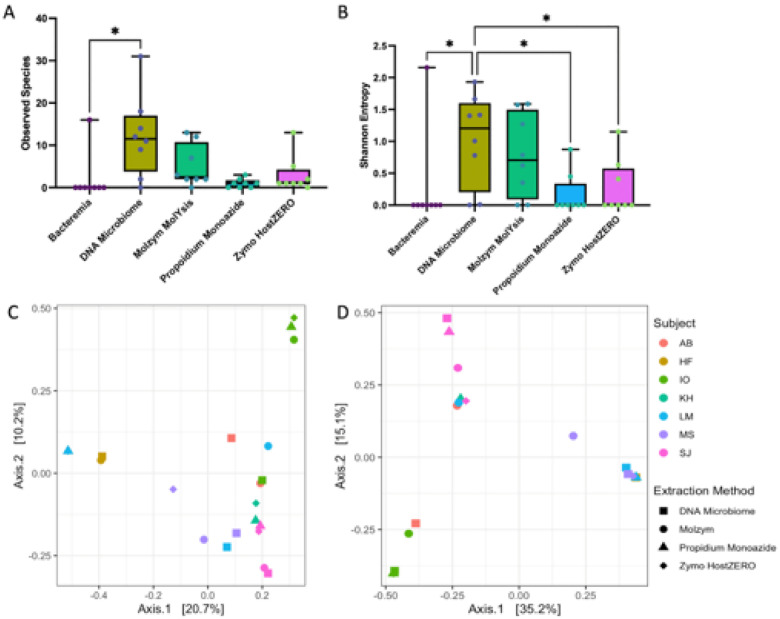
Extraction method and microbial diversity and composition (Shotgun metagenomics). Microbial diversity as measured by A) Observed Species (richness) and B) Shannon Entropy varied significantly by extraction method (Observed Species p=0.011, Shannon entropy, p=0.002, Friedman) with DNA Microbiome yielding significantly greater microbial diversity than other extraction methods (Observed Species DNA Microbiome vs. Bacteremia pairwise p=0.014, Shannon DNA Microbiome vs. all other methods (except Molzym MolYsis) pairwise p=0.014). Microbial species were identified via MetaPhlAn4. C) Microbial composition as measured by Jaccard or D) Bray-Curtis differed significantly by dog (Jaccard p=0.001, Bray-Curtis p=0.001, PERMANOVA), but not extraction method (Jaccard p=0.67, Bray-Curtis p=0.96, PERMANOVA). Urine samples extracted with Bacteremia contained little microbial DNA and did not produce reads that were assignable to a taxa by MetaPhlAn4. As such, Bacteremia samples were excluded from C) and D) and beta diversity testing.

To determine whether efficacy in host depletion translated to improved capture of the urobiome, we employed MetaPhlaAn4 and SingleM - computational tools used for profiling microbial communities from marker genes found in metagenomes. Urine microbial diversity varied significantly by extraction method (Fig. 6A, B, MetaPhlAn, Observed Species p = 0.011, Shannon entropy p = 0.002, Friedman), with DNA Microbiome yielding the greatest number of observed microbial species and significantly more species than all other extraction methods (all pairwise p = 0.014) except Molzym MolYsis. Urine microbial composition did not differ significantly by extraction method but did differ significantly by dog (Fig. 6C, D, MetaPhlAn4, By extraction method: Jaccard p = 0.67, Bray-Curtis p = 0.96; By dog: Jaccard p = 0.001, Bray Curtis p = 0.001, PERMANOVA), indicating that interindividual variation overwhelmed microbial community differences due to extraction method. SingleM largely recapitulated the MetaPhlAn results (**Fig. S9**).

**Figure 7:**
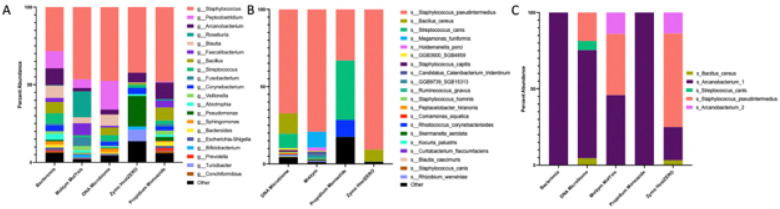
Top 20 microbial genera represented in urine samples. Relative abundances of the top 20 microbial genera identified in A) 16S rRNA sequencing of *unspiked* urine samples B) Shotgun metagenomic sequencing (MetaPhlAn4) of *spiked* urine samples, C) Metagenome-assembled-genomes (MAG) generated from *spikedurine* samples. The same urine samples were used for 16S and shotgun metagenomic sequencing. Across methods, *Staphylococcus (pseudintermedius), Bacillus* (*cereus*), *Streptococcus* (*canis*), and *Arcanobacterium* repeatedly emerge as abundant taxa. For 16S samples, ASVs were filtered to a minimum 0.5% abundance in at least 10% of samples. Host-spiked samples extracted with Bacteremia contained little microbial DNA and did not produce reads that were assignable to a taxa by MetaPhlAn4 and are thus excluded from B).

We then assessed the viability of performing genome-resolved metagenomics on low biomass urine samples. To do this, we assembled MAGs within each sample (Assembly metrics for each sample: **Fig. S10**). We generated a total of 26 unique MAGs: 11 were bacteria found in the ZymoBIOMICs Gut Microbiome Standard (**Table S3**), and five were derived from urine samples (Fig. 7); 10 were probable contaminants (**Table S5**). The five *E. coli* strains present in the standard assembled into a single MAG. The greatest number of urine-derived MAGs (n = 4) were identified in DNA Microbiome samples while three or fewer MAGs were identified in all other extraction methods. The total number of MAGs did not vary by extraction method (**Fig. S11**, p = 0.3, Friedman); although, fewer contaminant MAGs arose from DNA Microbiome samples as compared to other extraction methods (**Fig. S11**, overall p = 0.018, Friedman, no pairwise significant).

Next, we compared the microbial taxonomic profiles generated by 16S rRNA sequencing, shotgun metagenomic sequencing (MetaPhlAn4), and genome-resolved metagenomics (MAGs) (Fig. 7). Each method is fundamentally different and employs different reference databases for taxonomy assignment. However, all five urine-derived MAGs also appeared in the top twenty most abundant taxa in the shotgun metagenomics and 16S datasets. Notably, *Arcanobacterium* is not present in the MetaPhlAn4 reference database, but was identified in the shotgun metagenomic data through the SingleM reference database (**Fig S9**). Additional top 20 genera common between the metagenomics and 16S datasets include: *Peptacetobacter/Peptoclostridium spp*. and *Blautia spp*.

Finally, we compared our capture of the ZymoBIOMICs Gut Microbiome Standard community across extraction, sequencing, and bioinformatic methods (**Fig S12**). The Standard contained 21 microbial taxa including 18 bacterial strains, 1 Archaea, and 2 microbial eukaryotes at differing and biologically relevant abundances. Amongst the bacterial strains, there were 5 closely related strains of *E. coli*. In the 16S rRNA dataset, we were able to detect a total of 12/21 taxa, all of which were present at ≥0.1% abundance in the Standard. Expectedly, we did not detect the 2 microbial eukaryotes (which do not encode a 16S rRNA gene). We were also unable to differentiate the 5 *E. coli* strains in the Standard as this is not feasible with amplicon sequencing. We also did not detect the 4 taxa found at ≤0.01% abundance in the Standard *(Methanobrevibacter smithii, Salmonella enterica, Enterococcus faecalis, Clostridium perfringens)*. In the shotgun metagenomic data profiled using MetaPhlAn4, we detected a total of 14/21 taxa in the Standard including the 2 microbial eukaryotes. As with 16S rRNA sequencing, we were able to detect all taxa present at ≥0.1% abundance in the Standard and not able to detect the 4 taxa found at ≤0.01% abundance in the Standard. MetaPhlan4 did not distinguish the 5 *E. coli* strains. We were further able to assemble a total of 11 MAGs from the shotgun metagenomic data. This included all taxa at ≥1.5% abundance, excluding the eukaryote *Candida albicans,* which was found at 1.5% abundance but for which we were not able to assemble a MAG. We assembled a single *E. coli* MAG (rather than the expected 5 unique *E. coli* strains). The threshold we employed for MAG dereplication (99% ANI) did not allow us to distinguish between the 5 *E. coli* strains; therefore, as with our 16S rRNA data, we only detected “one” *E*. *coli* taxon. A higher ANI (99.9%) and a tool other than dRep would be required for strain differentiation. We were not able to assemble a MAG for *M. smithii* which was present at 0.1% abundance and detected in 16S rRNA and shotgun metagenomic sequencing. Across methods (16S rRNA, shotgun metagenomics, MAGs), samples extracted using Bacteremia and DNA Microbiome most closely matched the expected microbial taxonomic composition of the Standard (**Fig S12**).

### Functional Profiling of Urine Microbes

Relatively few studies have performed shotgun metagenomics in urine, and even fewer have generated MAGs [26], which has limited our understanding of the functional potential of the urobiome. In this study, as proof-of-concept, we mined the urine-derived MAGs for key functions. We first identified core metabolic pathways (e.g., glycolysis, citrate cycle) across all MAGs (**Fig. S13A**). Then we identified pathways associated with carbohydrate, nitrogen, acid, and alcohol metabolism. Specifically, we observed urea utilization in 2 of the MAGs: *Staphyocuccus pseudintermedius* and *Bacillus_A cerus*. (**Fig. S13B**).

**Figure 8:**
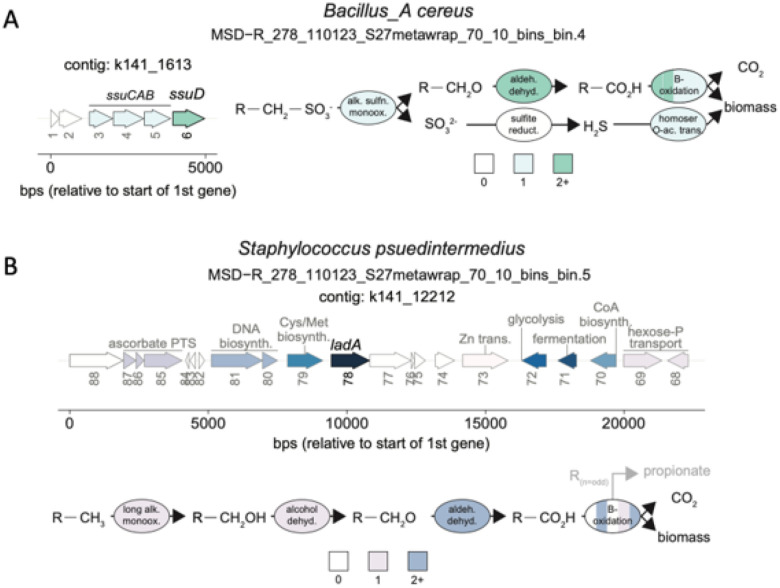
Metabolic potential of urine-associated MAGs. A) and B) feature reconstructed alkane metabolism in urine-associated MAGs. Shown are the regulon in which the predicted alkane metabolism gene occurs, as well as the reconstructed relevant pathway. For the depiction of the regulon, only up to ten neighboring genes on each side were included, and the coloring denotes arbitrary groupings with the gene responsible for alkane activation the darkest *(i.e., ssuDand ladB),* and genes that weren’t directly related to the predicted alkane metabolism colored grey. The numbers below the arrows indicate the gene number on the contig. For the depiction of the reconstructed alkane metabolism pathways, colors denote the number of genes that may be involved at each reaction, noting that for simplicity beta-oxidation has been summarized in one ellipse broken into five pieces. Further description of these results in **Supplementary Information 1.**

Next, we looked for microbial metabolic pathways associated with environmental chemical metabolism. There are a number of environmental chemicals (e.g., arsenic, polycyclic aromatic hydrocarbons) that have been linked to urinary tract diseases like bladder cancer [63]. The kidney filters many of these toxicants out of the blood and into the urine. Therefore, it is important to understand if and how urine microbes metabolize these chemicals and how that could impact disease risk. As such, we mined the urine MAGs for pathways associated with polycyclic aromatic hydrocarbon (PAH) and long-chain alkane degradation. PAHs and long-chain alkanes are common environmental pollutants produced during the combustion process and found in vehicle exhaust and industrial output [65–67]. We did not identify genes (> 80% gather cutoff) associated with PAH degradation but we did identify genes for long chain alkane utilization: *ladB* (91% of noise cutoff) in *Bacillus_A cereus* and *ladA* alpha (97% of trusted cutoff) in *Staphylococcus pseudintermedius*. Moreover, in *B. cereus,* we identified a full metabolic pathway starting with an alkanesulfonate monooxygenase *(ssuD)* that desulfonates organosulfonates to yield sulfite and an aldehyde (Fig. 8A). The presence of this pathway supports the possibility that *B. cereus* may be capable of utilizing a variety of hydrocarbons as potential carbon sources or electron donors. In *S. pseudintermedius,* we did not identify a complete metabolic pathway for long-chain alkane degradation, but the presence of alcohol and aldehyde dehydrogenase protein families suggest that long chain alkanes activated by *ladA* may be further oxidized by this organism (Fig. 8B). Taken together, these results suggest that urine-derived microbes can metabolize environmental chemicals, and that microbial metabolism merits further investigation in relation to urinary tract disease risk.

## Discussion

Studies of the urobiome are poised to reveal key insights in urinary tract health and disease; however, validation of approaches to profiling the urine microbial community are urgently needed. Here, we tested urine sampling volume and DNA extraction methods with host depletion using urine from healthy dogs. We identified a minimum urine volume threshold for for 16S rRNA and shotgun metagenomic sequencing, and we report on best host depletion methods for obtaining representative and reproducible microbial profiles. Finally, we demonstrate that MAG assembly is feasible in low-microbial, high-host biomass urine samples, and that even in this limited study, we were able to gain novel functional insights into urine-associated microbes.

In relation to urine volume, we observed that greater urine volumes (≥ 3 mL) resulted in improved microbial community capture, increased read depth (although not significant), reduced stochasticity / variability between samples, and reduced contaminant abundance (Fig. 1, 2, S5, S6). The largest urine volume tested in this study was 5 mL. It is possible that urine volumes > 5 mL may further increase recovery of rare taxa, though previous work has suggested that urine sample volume does not necessarily influence total biomass or sequencing depth [18]. Notably, one recent review anecdotally recommended 30 mL-50 mL of catheter-collected urine for 16S rRNA gene profiling [68]. Our study focused on mid-stream free catch urine, which can include microbes from the urethra or skin in addition to the bladder, and would therefore contain a higher microbial biomass than catheter-collected samples [9], which would be more representative of the bladder microbiota alone. Thus, it is reasonable to suggest that greater urine volumes would be advisable for urobiome studies that utilize catheter-collected urine; although, further study is warranted.

We next assessed the impact of DNA extraction methods with and without host depletion on multiple sample types (unspiked and host-spiked urine) and sequencing platforms (16S rRNA, shotgun metagenomics). In unspiked (low host biomass) urine, Bacteremia (no host depletion) and DNA Microbiome (host depletion) consistently yielded the greatest DNA concentrations and highest microbial diversity (16S rRNA) (Fig. 3, 4). Additionally, DNA Microbiome and Bacteremia-extracted samples were the most similar compositionally, and both of these methods most accurately captured the taxa and abundances of the ZymoBIOMICs Gut Microbiome Standard (**Fig. S12**). Notably, we were only able to reliably capture taxa that were found at ≥0.1% abundance in 16S rRNA and shotgun metagenomic data and generate MAGs from taxa found at ≥1.5% abundance. As observed in other studies, interindividual variation (between dogs) generally outweighed differences due to extraction method [18, 34]. However, when we employed phylogeny-aware metrics (Unweighted UniFrac), we saw significant differences in microbial composition by extraction method and by dog, suggesting that some host depletion methods bias microbial community profiles through preferential lysis of specific bacterial clades. Importantly, Bacteremia and DNA Microbiome have been identified as accurate and effective DNA extraction methods in other high-host, low-microbial biomass substrates (i.e., nasal swabs, vaginal swabs, urine, biopsies) [18, 69–71].

In host-spiked (high host biomass) urine samples, DNA Microbiome, Zymo and Molzym yielded the greatest percentage of microbial reads (22, 70, and 71% respectively) (shotgun metagenomics, Fig. 5). DNA Microbiome also recovered the greatest microbial diversity (MetaPhlAn4, Fig. 6). Notably, Bacteremia, with no host depletion, was not effective in capturing the microbial community in high host biomass urine. As in our 16S rRNA gene analysis, interindividual variation (between dogs) overwhelmed differences by extraction method, though we did not assess the MetaPhlAn4-profiled communities according to phylogenetic differences in composition. The Zymo HostZERO kit did not perform as well in this study as it has in studies on other substrates (respiratory, intestinal biopsy), suggesting that certain host depletion strategies may be differentially effective by substrate [21, 69]. Other technologies, not tested in this study, may also prove effective at microbial enrichment, including adaptive sequencing [72] and selective mechanical lysis [22].

Important insights have been revealed via read-level analysis of shotgun-sequenced urobiota. For example, in one study, shifts in microbial functional potential were observed in longitudinally collected urine samples of individuals with and without urinary tract symptoms.^4^ In another study, microbial virulence factor genes were linked to a distinct subset of individuals with urinary tract infections.^25^ A third study compared the urobiome of healthy individuals to calcium oxalate stone formers, and reported reduced abundances of genes associated with oxalate metabolism in the stone formers, suggesting that the urobiota may play a key role in urinary stone disease pathogeneis [26]. Whole-genome sequencing of cultured urine isolates has also revealed key insights: For example, genes enriched in strains of *E*. *faecalis* isolated from urine were not found in gut or blood isolates, suggesting unique adaptations for the urinary tract niche [24].

MAG generation offers advantages over read-level analyses and culture as it uniquely provides high-resolution information on specific microbes and their potential functions, without a dependence on culture [73]. Thus, we attempted *de novo* assembly of MAGs from our urine samples as proof-of-concept for genome-resolved metagenomics in urine. We assembled a total of five high quality (> 90% complete, < 10% contaminated), urine-derived microbial genomes: *B. cereus, S. pseudintermedius, S. canis,* and two unassigned *Arcanobacterium spp*. Notably, this study focused on mid-stream free catch urine samples which includes microbes from the bladder, urethra, and skin. Additionally, this study only included a small number of healthy individuals and was not designed to capture the breadth of urobiome diversity. To our knowledge, this is among the first reports of MAGs assembled from urine [26]. The MAGs we assembled have all been identified as members of the urobiota (or as potential uropathogens) in other studies [2, 34, 74].

Although the overall number of MAGs we recovered was low, we note that DNA Microbiome yielded a greater number of urine-derived MAGs and generally fewer contaminant MAGs as compared to all other extraction methods. Importantly, the fact that we were able to assemble 11 MAGs from contaminants (i.e. microbial DNA present in reagents and identifiable in negative control samples) highlights the need for rigorous negative controls as well as thorough bioinformatic decontamination to avoid spurious results. Well validated tools such as *decontam* [10, 36] as well as an awareness of common “kit-ome” taxa [56] are critical for microbiome studies of low biomass substrates.

After assembling MAGs, we went on to identify key functions in each MAG including core carbon and nitrogen metabolic pathways, urea metabolism, and environmental chemical degradation. We identified full urea-degrading complexes (ureABCEFGD) in 2 MAGs (*B. cereus* and *S. pseudintermedius)* in 3 the 7 dogs (**Fig. S13**). As urea is a major component of urine [75], the ability to metabolize urea may be a valuable function / adaptation for urine-associated microbes. As for environmental chemical degradation, there are well-established links between environmental chemical exposures and urinary tract diseases like bladder cancer [63, 76]. In fact, a recent meta-analysis reported that bacteria associated with PAH degradation were found at increased abundances in the urine of individuals with bladder cancer [2]. While we did not find evidence for microbial PAH degradation in this limited study on healthy dogs, we did find evidence for long-chain alkane degradation in 2 urine-derived MAGs (*B. cereus* and *S. pseudintermedius*) found in 3 of the 7 dogs. Long-chain alkanes are common environmental pollutants that result from industrial combustion processes [65, 77] and can be found in urine [78, 79]. Our findings novelly demonstrate that 2 urine-derived MAGs may degrade long-chain alkanes. This proof-of-concept study highlights the importance of understanding if and how host-associated microbes may be metabolizing environmental chemicals, so that we can then examine the potential impacts of this metabolism on host health or in diseases like bladder cancer [80–82].

## Conclusions

Key takeaways from this study:

1. Urine sample volumes of ^3^ 3 mL produced the most consistent urobiome profiles in dogs, which are a robust model for the human urobiome.
2. Microbial taxa found at ^3^ 0.1% abundance were reliably detected via 16S rRNA gene and shotgun metagenomic sequencing, but MAG assembly was only feasible at greater abundances (^3^ 1.5%), and strain differentiation in metagenomic data may require a higher ANI threshold than employed in this study (99% ANI was used in this study).
3. Generally, interindividual differences in urobiome profiles overwhelmed differences due to DNA extraction method.
4. In urine samples with low host biomass (unspiked), the QIAamp BiOstic Bacteremia kit (with no host depletion) yielded the greatest microbial DNA concentrations and highest microbial diversity (e.g. captured more / rarer urine taxa).
5. In urine samples with high host biomass (host-spiked), the QIAamp DNA Microbiome kit yielded the greatest microbial DNA concentrations, highest microbial diversity, and greatest number of identified metagenome-assembled genomes (MAGs), while effectively depleting host DNA.
6. MAG assembly is feasible but limited in urine samples. Maximizing urine volume to increase microbial reads would likely improve MAG recovery. Gene-based queries to assess functional potential of the urobiome are feasible with shotgun metagenomic data in the absence of MAG assembly; although, linking function (genes) to microbial species is more challenging with this approach.
7. Urine derived MAGs revealed evidence of urea and environmental chemical (long chain alkane) degradation, both of which are relevant for understanding how microbes live and adapt to the urine environment, as well as how they can potentially modulate environmental exposures in a way that could impact host health.

Urobiome research trails the study of other host-associated microbiomes [8], and continued optimization of urobiome profiling is critical to enable the mechanistic and functional insights necessary for understanding how these microbes impact host health.

## Data Availability

16S rRNA gene sequences are available on the Sequence Read Archive (SRA): BioProject PRJNA1109516. Shotgun metagenomic sequences are available at BioBroject PRJNA1123238. Sequence processing and analysis scripts are available on Github: https://github.com/zjlewis19/Evaluating-Urine-Volume-and-Host-Depletion-to-Enable-Shotgun-Metagenomics-of-the-Urobiome/blob/main/README.md

## Abbreviations

MAG: metagenome-assembled genome
EQUC: expanded quantitative urine culture
Bacteremia: QIAamp BiOstic Bacteremia DNA Kit
ASV: amplicon sequence variant
CTAC: canine thyroid adcenocarcinoma cell
Molzym MolYsis: Molzym MolYsis Complete5 DNA Isolation Kit
DNA Microbiome: Qiagen QIAamp DNA Microbiome Kit
Zymo HostZERO: Zymo HostZERO Microbial DNA Kit
PMA: propidium monoazide
IDI-GEMS: Infectious Diseases Institute – Genomics and Microbiology Solutions
GTDB-Tk: Genome Taxonomy Database – Toolkit
FDR: False discovery rate
PERMANOVA: Permutational analysis of variance
DRAM: Distilled and Refined Annotation of Metabolism
HMM: Hidden Markov Model
PAH: polycyclic aromatic hydrocarbon
PAHp: polycyclic aromatic hydrocarbon pathways
ANI: average nucleotide identity

## References

[1] Mrofchak R, Madden C, Evans MV, Kisseberth WC, Dhawan D, Knapp DW, Urine and fecal microbiota in a canine model of bladder cancer and comparison of canine and human urine microbiota. Life [Internet]. 2022 Dec 31 [cited 2023 Apr 2];15(1):1245–63. Available from: https://www.tandfonline.com/doi/full/10.1080/26895293.2022.2154858

[2] Bukavina L, Isali I, Ginwala R, Sindhani M, Calaway A, Magee D, Global Meta-analysis of Urine Microbiome: Colonization of Polycyclic Aromatic Hydrocarbon–degrading Bacteria Among Bladder Cancer Patients. Eur Urol Oncol [Internet]. 2023 Apr 1 [cited 2023 Jun 20];6(2):190–203. Available from: https://www.sciencedirect.com/science/article/pii/S2588931123000366 36868921 10.1016/j.euo.2023.02.004

[3] Pearce MM, Hilt EE, Rosenfeld AB, Zilliox MJ, Thomas-White K, Fok C, The Female Urinary Microbiome: a Comparison of Women with and without Urgency Urinary Incontinence. mBio [Internet]. 2014 Jul 8 [cited 2022 Sep 26];5(4):e01283–14. Available from: https://journals.asm.org/doi/10.1128/mBio.01283-14 25006228 10.1128/mBio.01283-14 PMC4161260

[4] Adu-Oppong B, Thanert R, Wallace MA, Burnham CAD, Dantas G. Substantial overlap between symptomatic and asymptomatic genitourinary microbiota states. Microbiome [Internet]. 2022 Jan 17 [cited 2023 Mar 29];10(1):6. Available from: 10.1186/s40168-021-01204-9 35039079 PMC8762997

[5] Worby CJ, Schreiber HL, Straub TJ, van Dijk LR, Bronson RA, Olson BS, Longitudinal multi-omics analyses link gut microbiome dysbiosis with recurrent urinary tract infections in women. Nat Microbiol [Internet]. 2022 May 2 [cited 2023 Jan 29];7(5):630–9. Available from: https://www.nature.com/articles/s41564-022-01107-x 35505248 10.1038/s41564-022-01107-x PMC9136705

[6] Stern JM, Moazami S, Qiu Y, Kurland I, Chen Z, Agalliu I, Evidence for a distinct gut microbiome in kidney stone formers compared to non-stone formers. Urolithiasis [Internet]. 2016 Oct [cited 2023 Jan 31];44(5):399–407. Available from: https://www.ncbi.nlm.nih.gov/pmc/articles/PMC8887828/27115405 10.1007/s00240-016-0882-9 PMC8887828

[7] Coffey EL, Gomez AM, Burton EN, Granick JL, Lulich JP, Furrow E. Characterization of the urogenital microbiome in Miniature Schnauzers with and without calcium oxalate urolithiasis. J Vet Intern Med [Internet]. 2022 [cited 2023 Mar 29];36(4):1341–52. Available from: https://onlinelibrary.wiley.com/doi/abs/10.1111/jvim.16482 35796316 10.1111/jvim.16482 PMC9308445

[8] Hilt EE, McKinley K, Pearce MM, Rosenfeld AB, Zilliox MJ, Mueller ER, Urine Is Not Sterile: Use of Enhanced Urine Culture Techniques To Detect Resident Bacterial Flora in the Adult Female Bladder. J Clin Microbiol [Internet]. 2014 Mar [cited 2023 Mar 29];52(3):871–6. Available from: https://www.ncbi.nlm.nih.gov/pmc/articles/PMC3957746/24371246 10.1128/JCM.02876-13 PMC3957746

[9] Karstens L, Asquith M, Caruso V, Rosenbaum JT, Fair D, Braun J, Community profiling of the urinary microbiota: Methodological considerations for low microbial biomass biological samples. Nat Rev Urol [Internet]. 2018 Dec [cited 2023 Mar 29];15(12):735–49. Available from: https://www.ncbi.nlm.nih.gov/pmc/articles/PMC6352978/30315209 10.1038/s41585-018-0104-z PMC6352978

[10] Karstens L, Asquith M, Davin S, Fair D, Gregory WT, Wolfe AJ, Controlling for Contaminants in Low-Biomass 16S rRNA Gene Sequencing Experiments. mSystems [Internet]. 2019 Jun 4 [cited 2023 Dec 4];4(4):e00290–19. Available from: https://www.ncbi.nlm.nih.gov/pmc/articles/PMC6550369/10.1128/mSystems.00290-19 PMC6550369 31164452

[11] Eisenhofer R, Minich JJ, Marotz C, Cooper A, Knight R, Weyrich LS. Contamination in Low Microbial Biomass Microbiome Studies: Issues and Recommendations. Trends Microbiol [Internet]. 2019 Feb 1 [cited 2023 Dec 4];27(2):105–17. Available from: https://www.cell.com/trends/microbiology/abstract/S0966-842X(18)30253-1 30497919 10.1016/j.tim.2018.11.003

[12] Price TK, Wolff B, Halverson T, Limeira R, Brubaker L, Dong Q, Temporal Dynamics of the Adult Female Lower Urinary Tract Microbiota. mBio [Internet]. 2020 Apr 21 [cited 2023 Mar 29];11(2):e00475–20. Available from: https://www.ncbi.nlm.nih.gov/pmc/articles/PMC7175091/32317321 10.1128/mBio.00475-20 PMC7175091

[13] Yoo JJ, Shin HB, Song JS, Kim M, Yun J, Kim Z, Urinary Microbiome Characteristics in Female Patients with Acute Uncomplicated Cystitis and Recurrent Cystitis. J Clin Med [Internet]. 2021 Mar 5 [cited 2023 Mar 29];10(5):1097. Available from: https://www.ncbi.nlm.nih.gov/pmc/articles/PMC7961880/33807946 10.3390/jcm10051097 PMC7961880

[14] Cheng Y, Chen Z, Gawthorne JA, Mukerjee C, Varettas K, Mansfield KJ, Detection of intracellular bacteria in exfoliated urothelial cells from women with urge incontinence. Pathog Dis [Internet]. 2016 Oct 1 [cited 2023 Dec 4];74(7):ftw067. Available from: 10.1093/femspd/ftw067 27402784

[15] Geerlings SE. Clinical Presentations and Epidemiology of Urinary Tract Infections. Microbiol Spectr [Internet]. 2016 Sep 9 [cited 2024 May 30];4(5):10.1128/microbiolspec.uti-0002-2012. Available from: https://journals.asm.org/doi/10.1128/microbiolspec.uti-0002-2012 27780014

[16] Flores C, Ling J, Loh A, Maset RG, Aw A, White IJ, A human urothelial microtissue model reveals shared colonization and survival strategies between uropathogens and commensals. Sci Adv [Internet]. 2023 Nov 10 [cited 2024 Jun 3];9(45):eadi9834. Available from: https://www.science.org/doi/10.1126/sciadv.adi9834 37939183 10.1126/sciadv.adi9834 PMC10631729

[17] Walker SP, Barrett M, Hogan G, Flores Bueso Y, Claesson MJ, Tangney M. Non-specific amplification of human DNA is a major challenge for 16S rRNA gene sequence analysis. Sci Rep [Internet]. 2020 Oct 1 [cited 2023 Dec 4];10(1):16356. Available from: https://www.nature.com/articles/s41598-020-73403-7 33004967 10.1038/s41598-020-73403-7 PMC7529756

[18] Karstens L, Siddiqui NY, Zaza T, Barstad A, Amundsen CL, Sysoeva TA. Benchmarking DNA isolation kits used in analyses of the urinary microbiome. Sci Rep [Internet]. 2021 Mar 17 [cited 2022 Dec 16];11:6186. Available from: https://www.ncbi.nlm.nih.gov/pmc/articles/PMC7969918/33731788 10.1038/s41598-021-85482-1 PMC7969918

[19] Marotz CA, Sanders JG, Zuniga C, Zaramela LS, Knight R, Zengler K. Improving saliva shotgun metagenomics by chemical host DNA depletion. Microbiome [Internet]. 2018 Feb 27 [cited 2023 Nov 8];6(1):42. Available from: 10.1186/s40168-018-0426-3 29482639 PMC5827986

[20] Duarte V da S, Porcellato D. Host DNA depletion methods and genome-centric metagenomics of bovine hindmilk microbiome. mSphere [Internet]. 2023 Dec 6 [cited 2023 Dec 11];0(0):e00470–23. Available from: https://journals.asm.org/doi/10.1128/msphere.00470-23 10.1128/msphere.00470-23 PMC10826364 38054728

[21] KimM, IiRCP, ShahVS, RossM, CormierJ, BaigA, Host DNA depletion on frozen human respiratory samples enables successful metagenomic sequencing for microbiome studies [Internet]. 2024 [cited 2024 Mar 26]. Available from: https://www.researchsquare.com/article/rs-3638876/v110.1038/s42003-024-07290-339609616

[22] Wu-Woods NJ, Barlow JT, Trigodet F, Shaw DG, Romano AE, Jabri B, Microbial-enrichment method enables high-throughput metagenomic characterization from host-rich samples. Nat Methods [Internet]. 2023 Nov [cited 2024 Mar 26];20(11):1672–82. Available from: https://www.nature.com/articles/s41592-023-02025-4 37828152 10.1038/s41592-023-02025-4 PMC10885704

[23] Du J, Khemmani M, Halverson T, Ene A, Limeira R, Tinawi L, Cataloging the phylogenetic diversity of human bladder bacterial isolates. Genome Biol [Internet]. 2024 Mar 21 [cited 2024 Mar 26];25(1):75. Available from: 10.1186/s13059-024-03216-8 38515176 PMC10958879

[24] Sharon BM, Arute AP, Nguyen A, Tiwari S, Reddy Bonthu SS, Hulyalkar NV, Genetic and functional enrichments associated with *Enterococcus faecalis* isolated from the urinary tract. Cooper VS, editor. mBio [Internet]. 2023 Dec 19 [cited 2024 Mar 26];14(6):e02515–23. Available from: https://journals.asm.org/doi/10.1128/mbio.02515-23 37962362 10.1128/mbio.02515-23 PMC10746210

[25] Moustafa A, Li W, Singh H, Moncera KJ, Torralba MG, Yu Y, Microbial metagenome of urinary tract infection. Sci Rep [Internet]. 2018 Mar 12 [cited 2024 Mar 26];8(1):4333. Available from: https://www.nature.com/articles/s41598-018-22660-8 29531289 10.1038/s41598-018-22660-8 PMC5847550

[26] Kachroo N, Monga M, Miller AW. Comparative functional analysis of the urinary tract microbiome for individuals with or without calcium oxalate calculi. Urolithiasis [Internet]. 2022 Jun 1 [cited 2024 Jun 24];50(3):303–17. Available from: 10.1007/s00240-022-01314-5 35234986 PMC11247624

[27] Shaffer M, Borton MA, McGivern BB, Zayed AA, La Rosa SL, Solden LM, DRAM for distilling microbial metabolism to automate the curation of microbiome function. Nucleic Acids Res [Internet]. 2020 Sep 18 [cited 2023 Dec 4];48(16):8883–900. Available from: 10.1093/nar/gkaa621 32766782 PMC7498326

[28] Knight R, Vrbanac A, Taylor BC, Aksenov A, Callewaert C, Debelius J, Best practices for analysing microbiomes. Nat Rev Microbiol [Internet]. 2018 Jul [cited 2023 Dec 4];16(7):410–22. Available from: https://www.nature.com/articles/s41579-018-0029-9 29795328 10.1038/s41579-018-0029-9

[29] Marques C, Belas A, Aboim C, Trigueiro G, Cavaco-Silva P, Gama LT, Clonal relatedness of Proteus mirabilis strains causing urinary tract infections in companion animals and humans. Vet Microbiol [Internet]. 2019 Jan 1 [cited 2023 Feb 1];228:77–82. Available from: https://www.sciencedirect.com/science/article/pii/S0378113518308733 30593384 10.1016/j.vetmic.2018.10.015

[30] Johnson JR, O’Bryan TT, Low DA, Ling G, Delavari P, Fasching C, Evidence of Commonality between Canine and Human Extraintestinal Pathogenic Escherichia coli Strains That Express papG Allele III. Infect Immun [Internet]. 2000 Jun [cited 2023 Feb 1];68(6):3327–36. Available from: https://www.ncbi.nlm.nih.gov/pmc/articles/PMC97593/10816481 10.1128/iai.68.6.3327-3336.2000 PMC97593

[31] Johnson JR, Clabots C, Kuskowski MA. Multiple-Host Sharing, Long-Term Persistence, and Virulence of Escherichia coli Clones from Human and Animal Household Members. J Clin Microbiol [Internet]. 2008 Dec [cited 2023 Dec 4];46(12):4078–82. Available from: https://www.ncbi.nlm.nih.gov/pmc/articles/PMC2593269/18945846 10.1128/JCM.00980-08 PMC2593269

[32] Knapp DW, Dhawan D, Ramos-Vara JA, Ratliff TL, Cresswell GM, Utturkar S, Naturally-Occurring Invasive Urothelial Carcinoma in Dogs, a Unique Model to Drive Advances in Managing Muscle Invasive Bladder Cancer in Humans. Front Oncol [Internet]. 2020 Jan 21 [cited 2022 Aug 22];9:1493. Available from: https://www.frontiersin.org/article/10.3389/fonc.2019.01493/full 32039002 10.3389/fonc.2019.01493 PMC6985458

[33] McGlynn A, Mrofchak R, Madan R, Madden C, Jahid MJ, Mollenkopf D, Longitudinal examination of urine pH, specific gravity, protein, culture, and antimicrobial resistance profiles in healthy dogs. J Vet Intern Med [Internet]. 2023 [cited 2023 Nov 20];37(6):2219–29. Available from: https://onlinelibrary.wiley.com/doi/abs/10.1111/jvim.16860 37682015 10.1111/jvim.16860 PMC10658500

[34] Mrofchak R, Madden C, Evans MV, Hale VL. Evaluating extraction methods to study canine urine microbiota. PLOS ONE [Internet]. 2021 Jul 9 [cited 2022 Sep 25];16(7):e0253989. Available from: https://journals.plos.org/plosone/article?id=10.1371/journal.pone.0253989 34242284 10.1371/journal.pone.0253989 PMC8270191

[35] Callahan BJ, McMurdie PJ, Rosen MJ, Han AW, Johnson AJA, Holmes SP. DADA2: High-resolution sample inference from Illumina amplicon data. Nat Methods [Internet]. 2016 Jul [cited 2023 Nov 6];13(7):581–3. Available from: https://www.nature.com/articles/nmeth.3869 27214047 10.1038/nmeth.3869 PMC4927377

[36] Davis NM, Proctor DM, Holmes SP, Relman DA, Callahan BJ. Simple statistical identification and removal of contaminant sequences in marker-gene and metagenomics data. Microbiome [Internet]. 2018 Dec 17 [cited 2023 Nov 6];6(1):226. Available from: 10.1186/s40168-018-0605-2 30558668 PMC6298009

[37] Benjamini Y, Krieger AM, Yekutieli D. Adaptive Linear Step-up Procedures That Control the False Discovery Rate. Biometrika [Internet]. 2006 [cited 2023 Nov 6];93(3):491–507. Available from: https://www.jstor.org/stable/20441303

[38] Hassan BB, Altstadt LA, Dirksen WP, Elshafae SM, Rosol TJ. Canine Thyroid Cancer: Molecular Characterization and Cell Line Growth in Nude Mice. Vet Pathol [Internet]. 2020 Mar 1 [cited 2024 Mar 26];57(2):227–40. Available from: 10.1177/0300985819901120 32081094

[39] Urinalysis and Body Fluids - F.A. Davis Company [Internet]. [cited 2024 Jan 17]. Available from: https://www.fadavis.com/product/urinalysis-and-body-fluids-strasinger-6

[40] Nadkarni MA, Martin FE, Jacques NA, Hunter N. Determination of bacterial load by real-time PCR using a broad-range (universal) probe and primers set. Microbiology [Internet]. 2002 [cited 2023 Nov 8];148(1):257–66. Available from: https://www.microbiologyresearch.org/content/journal/micro/10.1099/00221287-148-1-257 11782518 10.1099/00221287-148-1-257

[41] Szóstak N, Szymanek A, Hávranek J, Tomela K, Rakoczy M, Samelak-Czajka A, The standardisation of the approach to metagenomic human gut analysis: from sample collection to microbiome profiling. Sci Rep [Internet]. 2022 May 19 [cited 2023 Dec 11];12(1):8470. Available from: https://www.nature.com/articles/s41598-022-12037-3 35589762 10.1038/s41598-022-12037-3 PMC9120454

[42] Ohio Supercomputer Center. Columbus, OH: Ohio Supercomputer Center. [Internet]. Columbus, OH; 1987. Available from: http://osc.edu/ark:/19495/f5s1ph73

[43] Bolger AM, Lohse M, Usadel B. Trimmomatic: a flexible trimmer for Illumina sequence data. Bioinformatics [Internet]. 2014 Aug 1 [cited 2023 Nov 6];30(15):2114–20. Available from: 10.1093/bioinformatics/btu170 24695404 PMC4103590

[44] Woodcroft BJ. CoverM [Internet]. 2023 [cited 2023 Dec 11]. Available from: https://github.com/wwood/CoverM

[45] Blanco-Mfguez A, Beghini F, Cumbo F, McIver LJ, Thompson KN, Zolfo M, Extending and improving metagenomic taxonomic profiling with uncharacterized species using MetaPhlAn 4. Nat Biotechnol [Internet]. 2023 Nov [cited 2024 Mar 5];41(11):1633–44. Available from: https://www.nature.com/articles/s41587-023-01688-w 36823356 10.1038/s41587-023-01688-w PMC10635831

[46] Woodcroft BJ. wwood/singlem [Internet]. 2023 [cited 2023 Nov 6]. Available from: https://github.com/wwood/singlem

[47] Li D, Liu CM, Luo R, Sadakane K, Lam TW. MEGAHIT: an ultra-fast single-node solution for large and complex metagenomics assembly via succinct de Bruijn graph. Bioinformatics [Internet]. 2015 May 15 [cited 2023 Nov 6];31(10):1674–6. Available from: 10.1093/bioinformatics/btv033 25609793

[48] Gurevich A, Saveliev V, Vyahhi N, Tesler G. QUAST: quality assessment tool for genome assemblies. Bioinformatics [Internet]. 2013 Apr 15 [cited 2023 Nov 6];29(8):1072–5. Available from: 10.1093/bioinformatics/btt086 23422339 PMC3624806

[49] Uritskiy GV, DiRuggiero J, Taylor J. MetaWRAP–a flexible pipeline for genome-resolved metagenomic data analysis. Microbiome [Internet]. 2018 Sep 15 [cited 2023 Nov 6];6(1):158. Available from: 10.1186/s40168-018-0541-1 30219103 PMC6138922

[50] Kang DD, Li F, Kirton E, Thomas A, Egan R, An H, MetaBAT 2: an adaptive binning algorithm for robust and efficient genome reconstruction from metagenome assemblies. PeerJ [Internet]. 2019 [cited 2023 Nov 6];7. Available from: https://www.ncbi.nlm.nih.gov/pmc/articles/PMC6662567/10.7717/peerj.7359 PMC6662567 31388474

[51] Wu YW, Simmons BA, Singer SW. MaxBin 2.0: an automated binning algorithm to recover genomes from multiple metagenomic datasets. Bioinformatics [Internet]. 2016 Feb 1 [cited 2023 Nov 6];32(4):605–7. Available from: 10.1093/bioinformatics/btv638 26515820

[52] Alneberg J, Bjarnason BS, de Bruijn I, Schirmer M, Quick J, Ijaz UZ, Binning metagenomic contigs by coverage and composition. Nat Methods. 2014 Nov;11(11):1144–6. 25218180 10.1038/nmeth.3103

[53] Olm MR, Brown CT, Brooks B, Banfield JF. dRep: a tool for fast and accurate genomic comparisons that enables improved genome recovery from metagenomes through de-replication. ISME J [Internet]. 2017 Dec [cited 2023 Nov 6];11(12):2864–8. Available from: https://www.nature.com/articles/ismej2017126 28742071 10.1038/ismej.2017.126 PMC5702732

[54] Parks DH, Imelfort M, Skennerton CT, Hugenholtz P, Tyson GW. CheckM: assessing the quality of microbial genomes recovered from isolates, single cells, and metagenomes. Genome Res [Internet]. 2015 Jul [cited 2023 Nov 6];25(7):1043–55. Available from: https://www.ncbi.nlm.nih.gov/pmc/articles/PMC4484387/25977477 10.1101/gr.186072.114 PMC4484387

[55] Chaumeil PA, Mussig AJ, Hugenholtz P, Parks DH. GTDB-Tk: a toolkit to classify genomes with the Genome Taxonomy Database. Bioinformatics [Internet]. 2020 Mar 15 [cited 2023 Nov 6];36(6):1925–7. Available from: 10.1093/bioinformatics/btz848 PMC7703759 31730192

[56] Salter SJ, Cox MJ, Turek EM, Calus ST, Cookson WO, Moffatt MF, Reagent and laboratory contamination can critically impact sequence-based microbiome analyses. BMC Biol [Internet]. 2014 Nov 12 [cited 2023 Dec 11];12(1):87. Available from: 10.1186/s12915-014-0087-z 25387460 PMC4228153

[57] McMurdie PJ, Holmes S. phyloseq: An R Package for Reproducible Interactive Analysis and Graphics of Microbiome Census Data. PLOS ONE [Internet]. 2013 Apr 22 [cited 2024 Jun 28];8(4):e61217. Available from: https://journals.plos.org/plosone/article?id=10.1371/journal.pone.0061217 23630581 10.1371/journal.pone.0061217 PMC3632530

[58] Oksanen J, Simpson GL, Blanchet FG, Kindt R, Legendre P, Minchin PR, vegan: Community Ecology Package [Internet]. 2001 [cited 2024 Jun 28]. p. 2.6–6.1. Available from: https://CRAN.R-project.org/package=vegan

[59] Tidyverse [Internet]. [cited 2024 Jun 28]. Available from: https://www.tidyverse.org/

[60] Huang Y, Li L, Yin X, Zhang T. Polycyclic aromatic hydrocarbon (PAH) biodegradation capacity revealed by a genome-function relationship approach. Environ Microbiome [Internet]. 2023 Apr 30 [cited 2024 Feb 7];18(1):39. Available from: 10.1186/s40793-023-00497-7 37122013 PMC10150532

[61] Khot V, Zorz J, Gittins DA, Chakraborty A, Bell E, Bautista MA, CANT-HYD: A Curated Database of Phylogeny-Derived Hidden Markov Models for Annotation of Marker Genes Involved in Hydrocarbon Degradation. Front Microbiol [Internet]. 2022 Jan 7 [cited 2024 Apr 26];12. Available from: https://www.frontiersin.org/journals/microbiology/articles/10.3389/fmicb.2021.764058/full 10.3389/fmicb.2021.764058 PMC8767102 35069469

[62] Finn RD, Clements J, Eddy SR. HMMER web server: interactive sequence similarity searching. Nucleic Acids Res [Internet]. 2011 Jul 1 [cited 2024 Apr 26];39(Web Server issue):W29–37. Available from: https://www.ncbi.nlm.nih.gov/pmc/articles/PMC3125773/21593126 10.1093/nar/gkr367 PMC3125773

[63] Pelucchi C, Bosetti C, Negri E, Malvezzi M, La Vecchia C. Mechanisms of Disease: the epidemiology of bladder cancer. Nat Clin Pract Urol [Internet]. 2006 Jun [cited 2024 Apr 30];3(6):327–40. Available from: https://www.nature.com/articles/ncpuro0510 16763645 10.1038/ncpuro0510

[64] Bukavina L, Isali I, Ginwala R, Sindhani M, Calaway A, Magee D, Global Meta-Analysis of Urine Microbiome: Colonization of PAH-degrading bacteria among bladder cancer patients [Internet]. In Review; 2022 Sep [cited 2023 Feb 1]. Available from: https://www.researchsquare.com/article/rs-2003199/v1 10.1016/j.euo.2023.02.004 36868921

[65] Li J, Li K, Li H, Wang X, Wang W, Wang K, Long-chain alkanes in the atmosphere: A review. J Environ Sci [Internet]. 2022 Apr 1 [cited 2024 May 7];114:37–52. Available from: https://www.sciencedirect.com/science/article/pii/S1001074221002874 10.1016/j.jes.2021.07.021 35459500

[66] Chen J, Rodopoulou S, Strak M, de Hoogh K, Taj T, Poulsen AH, Long-term exposure to ambient air pollution and bladder cancer incidence in a pooled European cohort: the ELAPSE project. Br J Cancer [Internet]. 2022 Jun [cited 2024 Apr 8];126(10):1499–507. Available from: https://www.nature.com/articles/s41416-022-01735-4 35173304 10.1038/s41416-022-01735-4 PMC9090745

[67] Lima ALC, Farrington JW, Reddy CM. Combustion-Derived Polycyclic Aromatic Hydrocarbons in the Environment–A Review. Environ Forensics [Internet]. 2005 Jun 1 [cited 2024 May 7];6(2):109–31. Available from: 10.1080/15275920590952739

[68] Isali I, Helstrom EK, Uzzo N, Lakshmanan A, Nandwana D, Valentine H, Current Trends and Challenges of Microbiome Research in Bladder Cancer. Curr Oncol Rep [Internet]. 2024 [cited 2024 Apr 26];26(3):292–8. Available from: https://www.ncbi.nlm.nih.gov/pmc/articles/PMC10920447/38376627 10.1007/s11912-024-01508-7 PMC10920447

[69] Marchukov D, Li J, Juillerat P, Misselwitz B, Yilmaz B. Benchmarking microbial DNA enrichment protocols from human intestinal biopsies. Front Genet [Internet]. 2023 Apr 26 [cited 2024 Apr 28];14. Available from: https://www.frontiersin.org/journals/genetics/articles/10.3389/fgene.2023.1184473/full 10.3389/fgene.2023.1184473 PMC10169731 37180976

[70] Ahannach S, Delanghe L, Spacova I, Wittouck S, Van Beeck W, De Boeck I, Microbial enrichment and storage for metagenomics of vaginal, skin, and saliva samples. iScience [Internet]. 2021 Oct 16 [cited 2024 Apr 28];24(11):103306. Available from: https://www.ncbi.nlm.nih.gov/pmc/articles/PMC8571498/34765924 10.1016/j.isci.2021.103306 PMC8571498

[71] Ong CT, Boe-Hansen G, Ross EM, Blackall PJ, Turni C, Hayes BJ, Evaluation of Host Depletion and Extraction Methods for Shotgun Metagenomic Analysis of Bovine Vaginal Samples. Microbiol Spectr [Internet]. 2022 Apr 11 [cited 2024 Apr 29];10(2):e00412–21. Available from: https://journals.asm.org/doi/full/10.1128/spectrum.00412-21 10.1128/spectrum.00412-21 PMC9045270 35404108

[72] Marquet M, Zollkau J, Pastuschek J, Viehweger A, Schleußner E, Makarewicz O, Evaluation of microbiome enrichment and host DNA depletion in human vaginal samples using Oxford Nanopore’s adaptive sequencing. Sci Rep [Internet]. 2022 Mar 7 [cited 2024 Apr 29];12(1):4000. Available from: https://www.nature.com/articles/s41598-022-08003-8 35256725 10.1038/s41598-022-08003-8 PMC8901746

[73] Arikawa K, Ide K, Kogawa M, Saeki T, Yoda T, Endoh T, Recovery of strain-resolved genomes from human microbiome through an integration framework of single-cell genomics and metagenomics. Microbiome [Internet]. 2021 Oct 12 [cited 2024 Apr 29];9(1):202. Available from: 10.1186/s40168-021-01152-4 34641955 PMC8507239

[74] Bottone EJ. Bacillus cereus, a Volatile Human Pathogen. Clin Microbiol Rev [Internet]. 2010 Apr [cited 2024 Jun 18];23(2):382–98. Available from: https://www.ncbi.nlm.nih.gov/pmc/articles/PMC2863360/20375358 10.1128/CMR.00073-09 PMC2863360

[75] Mobley HL, Hausinger RP. Microbial ureases: significance, regulation, and molecular characterization. Microbiol Rev [Internet]. 1989 Mar [cited 2024 Apr 26];53(1):85–108. Available from: https://www.ncbi.nlm.nih.gov/pmc/articles/PMC372718/2651866 10.1128/mr.53.1.85-108.1989 PMC372718

[76] Knapp DW, Dhawan D, Ruple A, Cooper BR, Zhang M, Liu D, Association between cigarette smoke exposure and urinary bladder cancer in Scottish terriers in a cohort study. Vet J [Internet]. 2024 Feb 1 [cited 2024 Jan 16];303:106044. Available from: https://www.sciencedirect.com/science/article/pii/S1090023323000953 38000695 10.1016/j.tvjl.2023.106044

[77] Glasius M, Thomsen D, Wang K, Iversen LS, Duan J, Huang RJ. Chemical characteristics and sources of organosulfates, organosulfonates, and carboxylic acids in aerosols in urban Xi’an, Northwest China. Sci Total Environ [Internet]. 2022 Mar 1 [cited 2024 May 7];810:151187. Available from: https://www.sciencedirect.com/science/article/pii/S0048969721062653 34756911 10.1016/j.scitotenv.2021.151187

[78] Llambrich M, Brezmes J, Cumeras R. The untargeted urine volatilome for biomedical applications: methodology and volatilome database. Biol Proced Online [Internet]. 2022 Dec 1 [cited 2024 May 6];24(1):20. Available from: 10.1186/s12575-022-00184-w 36456991 PMC9714113

[79] Bouatra S, Aziat F, Mandal R, Guo AC, Wilson MR, Knox C, The Human Urine Metabolome. PLOS ONE [Internet]. 2013 Sep 4 [cited 2024 May 7];8(9):e73076. Available from: https://journals.plos.org/plosone/article?id=10.1371/journal.pone.0073076 24023812 10.1371/journal.pone.0073076 PMC3762851

[80] Sowada J, Schmalenberger A, Ebner I, Luch A, Tralau T. Degradation of benzo[a]pyrene by bacterial isolates from human skin. FEMS Microbiol Ecol [Internet]. 2014 [cited 2023 Aug 23];88(1):129–39. Available from: https://onlinelibrary.wiley.com/doi/abs/10.1111/1574-6941.12276 24372170 10.1111/1574-6941.12276

[81] Sowada J, Lemoine L, Schön K, Hutzler C, Luch A, Tralau T. Toxification of polycyclic aromatic hydrocarbons by commensal bacteria from human skin. Arch Toxicol [Internet]. 2017 [cited 2023 Aug 23];91(6):2331–41. Available from: https://www.ncbi.nlm.nih.gov/pmc/articles/PMC5429354/28378121 10.1007/s00204-017-1964-3 PMC5429354

[82] Van De Wiele T, Vanhaecke L, Boeckaert C, Peru K, Headley J, Verstraete W, Human Colon Microbiota Transform Polycyclic Aromatic Hydrocarbons to Estrogenic Metabolites. Environ Health Perspect [Internet]. 2005 Jan [cited 2023 Sep 12];113(1):6–10. Available from: https://ehp.niehs.nih.gov/doi/10.1289/ehp.7259 15626640 10.1289/ehp.7259 PMC1253702

[83] Reichenbecher W, Murrell JC. Linear alkanesulfonates as carbon and energy sources for gram-positive and gram-negative bacteria. Arch Microbiol [Internet]. 1999 May 18 [cited 2024 May 7];171(6):430–8. Available from: http://link.springer.com/10.1007/s002030050730 10369899 10.1007/s002030050730

[84] Jimenez-Diaz L, Caballero A, Segura A. Pathways for the Degradation of Fatty Acids in Bacteria. In: Rojo F, editor. Aerobic Utilization of Hydrocarbons, Oils and Lipids [Internet]. Cham: Springer International Publishing; 2017 [cited 2024 May 7]. p. 1–23. Available from: 10.1007/978-3-319-39782-5_42-1

